# Cough-generated aerosols of *Pseudomonas aeruginosa* and other Gram-negative bacteria from patients with cystic fibrosis

**DOI:** 10.1136/thx.2008.112466

**Published:** 2009-07-01

**Authors:** C E Wainwright, M W France, P O’Rourke, S Anuj, T J Kidd, M D Nissen, T P Sloots, C Coulter, Z Ristovski, M Hargreaves, B R Rose, C Harbour, S C Bell, K P Fennelly

**Affiliations:** 1 Royal Children’s Hospital and Health Service District, Brisbane, Australia; 2 Department of Paediatrics and Child Health, University of Queensland, Brisbane, Australia; 3 Thoracic Medicine, The Prince Charles Hospital, Brisbane, Australia; 4 Queensland Institute of Medical Research, Brisbane, Australia; 5 Qpid Laboratory, Sir Albert Sakzewski Virus Research Centre, Herston, Australia; 6 Pathology Queensland, Herston, Australia; 7 School of Medicine, University of Queensland, Brisbane, Australia; 8 Infectious Diseases, The Prince Charles Hospital, Brisbane, Australia; 9 IHBI Queensland University Technology, Brisbane, Australia; 10 Department of Infectious Diseases, University of Sydney, Sydney, Australia; 11 UMDNJ-New Jersey Medical School, New Jersey, USA

## Abstract

**Background:**

*Pseudomonas aeruginosa* is the most common bacterial pathogen in patients with cystic fibrosis (CF). Current infection control guidelines aim to prevent transmission via contact and respiratory droplet routes and do not consider the possibility of airborne transmission. It was hypothesised that subjects with CF produce viable respirable bacterial aerosols with coughing.

**Methods:**

A cross-sectional study was undertaken of 15 children and 13 adults with CF, 26 chronically infected with *P aeruginosa*. A cough aerosol sampling system enabled fractioning of respiratory particles of different sizes and culture of viable Gram-negative non-fermentative bacteria. Cough aerosols were collected during 5 min of voluntary coughing and during a sputum induction procedure when tolerated. Standardised quantitative culture and genotyping techniques were used.

**Results:**

*P aeruginosa* was isolated in cough aerosols of 25 subjects (89%), 22 of whom produced sputum samples. *P aeruginosa* from sputum and paired cough aerosols were indistinguishable by molecular typing. In four cases the same genotype was isolated from ambient room air. Approximately 70% of viable aerosols collected during voluntary coughing were of particles ⩽3.3 μm aerodynamic diameter. *P aeruginosa*, *Burkholderia cenocepacia*, *Stenotrophomonas maltophilia* and *Achromobacter xylosoxidans* were cultivated from respiratory particles in this size range. Positive room air samples were associated with high total counts in cough aerosols (p = 0.003). The magnitude of cough aerosols was associated with higher forced expiratory volume in 1 s (r = 0.45, p = 0.02) and higher quantitative sputum culture results (r = 0.58, p = 0.008).

**Conclusion:**

During coughing, patients with CF produce viable aerosols of *P aeruginosa* and other Gram-negative bacteria of respirable size range, suggesting the potential for airborne transmission.

*Pseudomonas aeruginosa* is the most common bacterial pathogen in patients with cystic fibrosis (CF).^1^ The prevalence of chronic *P aeruginosa* increases with age, and is a major predictor of mortality and morbidity.^2^ It is unclear to what extent cross-infection of *P aeruginosa* between patients with CF occurs.^3^ ^4^ While siblings with CF can harbour the same *P aeruginosa* strain, it was thought until recently that most patients had their own individual strain acquired from the environment.^5^ With the advent of molecular typing methods there is now convincing evidence of clonal *P aeruginosa* infection in patients attending some paediatric and adult CF centres.^6^ ^7^ *P aeruginosa* has been cultured from soap holders held at up to 40 cm from the mouth of coughing patients with CF, supporting large respiratory droplet spread.^8^ The exact mechanisms involved in the spread of bacteria in CF clinics remain unclear.^6^ ^7^ Two studies have isolated clonal *P aeruginosa* during environmental air sampling up to 10 m from patients with CF infected with clonal strains while performing physiotherapy and lung function testing, suggesting the potential of person-to-person spread via the airborne route.^9^ ^10^

Current guidelines for infection control for patients with CF recommend only contact and droplet precautions—that is, focusing on hand hygiene and avoiding close contact between patients with CF who are advised to maintain a distance of at least 1 m from other patients.^11^ ^12^ It is possible that airborne transmission of *P aeruginosa*, *Burkholderia cepacia* complex and other bacteria may occur in addition to other modes of transmission.^13^ The relative contribution of the airborne route may be opportunistic in nature and occur in certain circumstances, such as in enclosed spaces with favourable ambient temperature and humidity as may occur in hospital, clinic and congregate settings.

Particle size distribution of aerosols is a key determinant for both deposition in the respiratory tract and for the ability of particles to remain airborne. To our knowledge, the particle size distribution of aerosols from patients with CF has never been reported. We hypothesised that, during voluntary coughing and during sputum induction, subjects with CF produce viable bacterial aerosols that are respirable. To test this hypothesis we modified a cough aerosol sampling system (CASS) recently developed to measure cough-generated aerosols from patients with *Mycobacterium tuberculosis*.^14^

Our primary aim was to determine the concentration and particle size distribution of cough aerosols containing culturable *P aeruginosa* and other Gram-negative bacteria from children and adults with CF. We also sought to determine whether concentrations of cough aerosols detected were related to clinical parameters and clonality of *P aeruginosa* strains.

## Methods

### Subjects

Subjects with CF were recruited from both the inpatient and outpatient services at the Royal Children’s Hospital and The Prince Charles Hospital in Brisbane, Australia. Inclusion criteria were age >9 years, a confirmed diagnosis of CF and culture of *P aeruginosa* or *B cepacia* complex from sputum on at least one occasion within the previous 12 months. Exclusion criteria included known pregnancy, pneumothorax within the previous 6 months, history of cough syncope or vomiting associated with coughing. After the first subject experienced recurrence of mild haemoptysis during the cough study, we excluded those with haemoptysis in the previous 7 days. Subjects were excluded from hypertonic saline inhalation if there was a history of intolerance of hypertonic saline, presence of asthma symptoms or a forced expiratory volume in 1 s (FEV_1_) ⩽40% predicted and no previous trials of hypertonic saline. Subjects were asked to withhold all nebulised therapy for 12 h prior to testing.

### Cough aerosol sampling system (CASS)

The equipment used was a modification of that developed previously.^14^ In brief, a subject coughs through a mouthpiece connected to afferent tubing into a chamber whereupon a vacuum pump draws exhaled air and generated respiratory particles through one of two Anderson six-stage impactors. Each stage has 400 holes of decreasing diameter through which appropriately-sized aerosolised particles will penetrate and deposit on an agar plate. A “settle plate” of the same agar was placed inside the chamber to capture larger droplets. Larger particles (droplets) would be expected to deposit in the afferent limb tubing, the settle plate and the walls of the chamber. Additional details are provided in the online data supplement.

### CASS protocol

The Andersen impactors were loaded with agar plates at room temperature. The tubing from the vacuum pump was attached to the port for the first six-stage impactor in the CASS. After the first session of coughing, the tubing was moved to the second sampler. All unused ports were occluded with plastic tape.

Subjects were instructed to cough into the CASS as frequently and as strongly as was comfortable for 5 min. At the onset of coughing the timer (set for 5 min) controlling the power to the vacuum pump was started. Cough strength was assessed as strong, moderate or weak and cough frequency was assessed quantitatively.

If hypertonic saline could be tolerated, the first sampling was done during voluntary coughing and the second 5-minute sample was collected during inhalation of 5 ml 4.5% saline delivered by a handheld ultrasonic nebuliser (Microneb Allersearch distributed by Becton Dickinson, North Ryde, Australia). Subjects were pretreated with albuterol metered dose inhaler (88 μg per puff), 4 puffs via spacer (Volumatic, Allen & Hanburys, UK). If hypertonic saline was not considered safe, sampling was done with the subject using tidal breathing for 5 min. Sputum samples were collected if produced.

### Clinical parameters

#### Pulmonary function testing

Forced expiratory volume in 1 s (FEV_1_) and forced vital capacity (FVC) were obtained according to standard guidelines prior to the cough study.^15^ Respiratory muscle strength was assessed using maximum inspiratory pressure (MIP) and maximum expiratory pressure (MEP) (Morgan Pmax) at the paediatric centre and using a Micro Medical Respiratory Pressure Meter (Micro Medical, Rochester, UK) at the adult centre. 

#### Other

Age, gender, presence of current exacerbation of disease, height, weight and body mass index were recorded.

### Room air sampling and air exchange

Using a centrifugal air sampler, two samples were obtained before each cough aerosol study: one during the subjects’ performance of spirometry and one during the cough aerosol study. The indoor air temperature and relative humidity were measured with a thermohygrometer (Rotronic HygroPalm 2, Rotronic Instrument Corp, Huntington, New York, USA) at the beginning of each study. Effective air exchange rates in the consultation rooms used for CASS testing and in the pulmonary function laboratory at the adult centre were determined using carbon dioxide as a tracer gas. Further details are provided in the online supplement.

### Microbiology

#### CASS aerosol samples and chamber settle plate

Cultures were performed using chocolate bacitracin (300 μg/ml) agar in aerobic conditions at 35°C. After 48 h and 72 h incubation, a colony forming unit (CFU) count was performed on each plate including individual colonial *P aeruginosa* morphotypes and the combined total CFU count of *P aeruginosa* and other Gram-negative bacteria. Following presumptive screening (characteristic colonial appearance, presence of oxidase and growth at 42°C), the identity of each *P aeruginosa* isolate was confirmed by species-specific *oprL* gene PCR.^16^ Other non-fermenting Gram-negative bacteria detected throughout the study were identified using a combination of API 20NE (bioMerieux), amplified rDNA restriction analysis (ARDRA) and *recA*-based PCR analysis.^17^ ^18^

Each Andersen sampler stage contains 400 holes and each CFU is regarded as the result of an infectious particle within a specific size range impacting on the agar. Colony counts exceeding 400 have been interpreted in two ways: an accepted “positive-hole” correction model taking into account the probability of multiple hits through each hole and a conservative model of a maximum count of 400 only.^19^ ^20^ The total sum of *P aeruginosa* or *B cepacia* complex colonies counted (total count) in all the Andersen stages for 5 min of voluntary coughing and for 5 min hypertonic saline study or tidal breathing was calculated, as was the sum of the colonies from stages 4, 5 and 6 (<3.3 μm, termed “small aerosol fraction”).

#### Sputum samples, afferent limb cultures and air samples

Standard quantitative culture methods were used.^21^ For air samples and afferent limb cultures, only Gram-negative non-fermentative bacteria were assessed. Isolates were identified as above with molecular strain typing of *P aeruginosa* isolates. Further details are provided in the online data supplement.

### Analysis of data

Counts for individual components and the totals for Andersen stages 1–6 (total) and for Andersen stages 4–6 (small fraction) were logarithmically transformed before analysis to correct for skewness. Means and 95% confidence limits (95% CI) were back transformed from log to linear scales for presentation. The paired differences between counts during voluntary coughing and each of the hypertonic and tidal breathing studies were analysed by paired *t* tests and mean differences were also back transformed from log to linear scales to calculate the ratios of counts during voluntary coughing and each of the hypertonic and tidal breathing studies. Correlation coefficients were estimated between logarithmically transformed total counts and clinical and demographic factors where available for all subjects. The Fisher exact test was used for the association between positive air samples and high total counts. All reported p values are two-sided. Linear regression was used to estimate the slope of the relationship between FEV_1_ and total count. All analyses were performed with SPSS software Version 15.

## Results

### CASS studies

Twenty-eight subjects (15 children, 13 adults) were consecutively recruited and completed 5 min of voluntary coughing. Twenty subjects were administered nebulised hypertonic saline and seven subjects had measurements during tidal breathing. One subject performed the voluntary cough only. Thirteen subjects were studied during a pulmonary exacerbation (table 1).

**Table 1:** Demographic and baseline clinical factors of study subjects

	Children(n = 15)	Adults(n = 13)	All(n = 28)
Median (range) age (years)	13.5 (9.9–16.6)	25.8 (18.8–48.8)	16.4 (9.9–48.8)
Gender (M/F)	8/7	9/4	17/11
Current exacerbation	7 (47%)	6 (46%)	13 (46%)
Mean (SD) BMI (kg/m^2^)	17.9 (2.5)	22.6 (3.7)	20.1 (3.9)
Mean (SD) Z score for weight	−0.6 (0.9)		
Mean (SD) Z score for height	−0.5 (1.2)		
Mean (SD) FEV_1_ (% predicted)	67.0 (22.5)	52.4 (19.6)	60.2 (22.1)
Mean (SD) FVC (% predicted)	77.0 (19.6)	70.7 (15.4)	74.1 (17.7)
Mean (SD) peak flow (l/s)	4.6 (2.2)	6.1 (1.9)	5.4 (2.2)
Mean (SD) MIP (cm H_2_O)	82.8 (32.8)	101.0 (30.1)	91.1 (32.3)
Mean (SD) MEP (cm H_2_O)	113.1 (42.9)	103.6 (29.5)	109.1 (37.4)

BMI, body mass index; FEV_1_, forced expiratory volume in 1 s; FVC, forced vital capacity; MEP, maximum expiratory pressure; MIP, maximum inspiratory pressure.

### Sputum microbiology

In the 12 months before the study, 27 subjects had sputum that cultured positive for *P aeruginosa* and one subject had cultured *B cenocepacia* (table 2). Of the 27 patients with *P aeruginosa* infection, all adults (n = 12) and 14 children had chronic infection based on the Leeds criteria.^22^ One child had recently cleared a new infection with *P aeruginosa* following an eradication course of antibiotic therapy and cultured normal respiratory flora from a sputum sample collected on the day of testing. The patient with *B cenocepacia* had chronic infection based on the Leeds criteria (table 2).^22^ On the study day, 23 subjects provided expectorated sputum samples. Of these, one subject grew *B cenocepacia* as expected and *P aeruginosa* was cultured in 21. In six subjects *Staphylococcus aureus* was cultured and in two methicillin-resistant *S aureus* was cultured. Other organisms cultured from sputum included α-haemolytic streptococci, *Aspergillus* species and yeasts. Molecular strain typing demonstrated a common clone corresponding to the previously described Australian Epidemic Strain 2 (AES2) in 16 subjects (6 adults, 10 children) and 5 had unique strains (4 adults, 1 child) (table 2).^23^

**Table 2:** Microbiology of sputum and CASS samples

	Infection status prior to study	Expectorated sputum(n = 23)	CASS study Voluntary cough (n = 28)/hypertonic saline (n = 20)	Tidal study(n = 7)
*P aeruginosa* not isolated	Cleared *P aeruginosa* (n = 1)	1	3	4
*P aeruginosa* isolated	Chronic *P aeruginosa* (n = 12 adults, n = 14 children)	21	25*	3
	*P aeruginosa* (unique)	5	7	1
	*P aeruginosa* (AES2)	16	18	2
*B cenocepacia* isolated	Chronic *B cenocepacia* (n = 1)	1	1	0

AES2, Australian Epidemic Strain 2; CASS, cough aerosol sampling system.

*Five subjects also cultured additional Gram-negative bacteria in cough aerosols (4 *Stenotrophomonas maltophilia* and 1 *Achromobacter xylosoxidans*).

### CASS microbiology

Of the 28 subjects, 25 had cough aerosols that grew *P aeruginosa*. One subject cultured *P aeruginosa* from cough aerosols only with the hypertonic saline study and not from voluntary coughing. One subject cultured *B cenocepacia* from cough aerosols. Two subjects had no Gram-negative bacteria cultured from cough aerosols. In five subjects with cough aerosols with *P aeruginosa*, additional Gram-negative bacteria were co-cultured (*Stenotrophomonas maltophilia* in four and *Achromobacter xylosoxidans* in one, table 2). Two of the subjects who co-cultured *S maltophilia* did not produce sputum and sputum culture was negative for *S maltophilia* for the other two. Three of the four subjects cultured *S maltophilia* intermittently from sputum at other times. The subject with *A xylosoxidans* in the cough aerosol culture did not culture the organism in the sputum sample on this occasion, although the subject was known to be chronically infected with this organism which had been cultured repeatedly from previous sputum samples.

**Figure 1:**
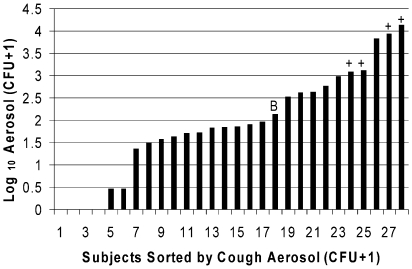
Distribution of total corrected voluntary cough aerosols. B, subject with *Burkholderia cenocepacia*; CFU, colony forming unit; +, positive ambient air samples isolated.

The corrected total count of CFUs obtained from generated aerosols varied widely among subjects and was log normally distributed (voluntary cough: range 0–13 485 CFU, fig 1). All subjects but one who cultured *P aeruginosa* in sputum also cultured *P aeruginosa* of identical genotype in the CASS cough aerosols. The total count from sputum correlated with the total corrected count for voluntary coughing from the aerosols (r = 0.58, p = 0.008). Three of the seven subjects who had tidal breathing studies had positive CASS aerosol cultures, with *P aeruginosa* cultured in low numbers (total aerosol counts from tidal breathing 1, 5 and 137 CFU).

### Settle plate and air sampling microbiology

The chamber settle plate and afferent limb equipment was not changed between the two components of the study for individual subjects with quantitative culture, reflecting large droplet deposition for both components of the study combined. The mean total count for the settle plate was 6 CFU (95% CI 3 to 14). The mean total count for the afferent limb was 56 CFU/ml wash fluid (95% CI 10 to 303).

Mean (SE) air exchange rates ranged between 9.77 (0.06) and 19.40 (0.70) exchanges per hour in the testing rooms. A total of 101 air samples were collected before and during testing. Sixteen samples cultured unique strains of *P aeruginosa* during testing of 14 patients. The unique strains isolated did not match any sputum or CASS isolates. Five air samples cultured AES2 strain during testing of four subjects with AES2 strain of *P aeruginosa*. For these four subjects, sputum, cough aerosol and air samples all cultivated the same strain. Three of the AES2 positive air samples were collected during pulmonary function testing and two during background testing in the CASS study rooms. Positive air samples were associated with a high concentration in cough aerosols. If only subjects with AES2 were considered, four out of five subjects with total cough aerosol counts >1000 CFU had positive air samples and no subjects out of the 10 with lower total CFU counts had positive air samples (p = 0.003). Temperature and humidity did not vary significantly between study sites or study days at each site (data not shown).

### CASS microbiology: voluntary cough, hypertonic cough and tidal breathing

**Figure 2:**
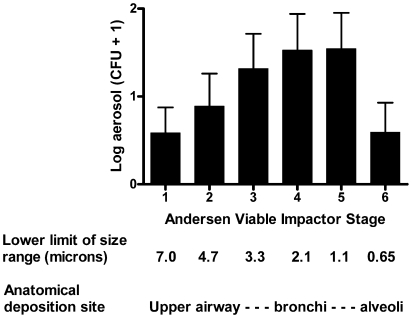
Particle size distribution of logarithmic corrected total cough aerosol counts in colony forming units (CFU) with 95% confidence intervals during voluntary coughing according to Andersen stage.

The infective particle size distribution of cough aerosols of *P aeruginosa* or *B cenocepacia* during voluntary coughing is shown in fig 2. Using the corrected total counts, 71.8% of particles (95% CI 66.8% to 76.8%) containing culturable aerosol isolated from voluntary coughing were on Andersen stages 4, 5 and 6 of the Andersen samplers (small aerosol fraction ⩽3.3 μm). The conservative model gave similar results with 69.9% (95% CI 64.6% to 75.4%) in the small aerosol fraction. Mean total corrected counts were much lower during tidal breathing (2, 95% CI −0.5 to 15) than during voluntary coughing (85, 95% CI 28 to 238; p<0.001) or hypertonic saline (68, 95% CI 21 to 215). There was no significant difference in total corrected counts between voluntary coughing and hypertonic saline (p = 0.12). The pattern of differences was unaffected by using the conservative model (data not shown).

### CASS microbiology: clinical correlates

**Figure 3:**
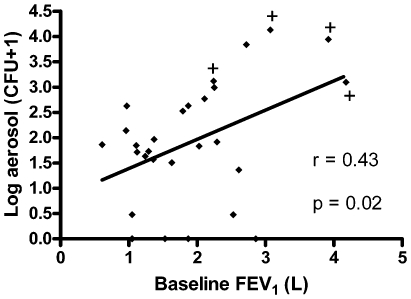
Correlation of baseline forced expiratory volume in 1 s (FEV_1_) with logarithmic total corrected count from cough aerosols during voluntary coughing. CFU, colony forming unit; +, positive ambient air samples isolated.

FEV_1_ correlated with the total corrected count from the voluntary cough aerosol (r = 0.45, p = 0.019; fig 3) and also with the corrected small aerosol fraction (r = 0.45, p = 0.018). Similarly, FEV_1_ correlated with the conservative total count (r = 0.39, p = 0.044) and conservative small aerosol fraction (r = 0.39, p = 0.047) during voluntary coughing. There was no significant association between cough aerosol CFU counts and any other clinical factor including gender, age, testing at paediatric or adult centre, current exacerbation status, FVC, MIP, MEP, percentage predicted FEV_1_, presence of clonal *P aeruginosa*, quality or actual number of coughs counted (data not shown). There was a trend for an association of peak expiratory flow (r = 0.36, p = 0.079) and of body mass index (r = 0.37, p = 0.058) with total corrected count for voluntary coughing.

## Discussion

This is the first study to report the magnitude, variability and particle size distribution of culturable aerosols of Gram-negative bacteria produced by coughing in patients with CF. Although there is evidence of culturable Gram-negative bacteria in the large droplets within the afferent tubing and settle plates in the cough chamber, a large proportion of culturable particles were found to be in a size range that is likely to deposit in the lower respiratory tract. Genetically indistinguishable bacteria were identified in expectorated sputum and in the cough-generated aerosols, and in four experiments the same organisms were also isolated from the ambient room air. This supports the assertion that the sources of the bacteria are the patients rather than the hospital or nearby environment.

Aerosolisation of respiratory tract particles during coughing and sneezing and even during tidal breathing is a well recognised phenomenon associated with the spread of many infections including measles, influenza and tuberculosis.^24^ ^25^ ^26^ The majority of respiratory pathogens have been thought to be spread by large droplets that settle within an approximate 1 m range of an individual, providing a low risk of airborne infection. Infection control practices for most CF centres reflect recently published infection control guidelines suggesting that patients should maintain a distance of at least 1 m to reduce the risk of cross-infection.^12^ The risk of acquisition of infection from respiratory aerosols is complex and probably relates to the pathogen type, concentration of the organism in the aerosol, the susceptibility of exposed individuals and the environment (air movement, relative humidity, temperature, etc). Limited studies have examined particle size distribution of respiratory aerosols and most have reported large droplet formation, predominantly particles with a diameter of >8 μm.^27^ ^28^ More recently, Papineni and Rosenthal reported that 85% of particles were <1 μm and that coughing produced more aerosol particles than did breathing or talking.^29^ The first published study to use a CASS examined patients with tuberculosis and, like our study, found that most of the respiratory particles were <3.3 μm.^14^ Our study shows that patients with CF produce culturable aerosols in a wide range of particle sizes including both respiratory droplets and infectious droplet nuclei. We have shown this predominantly for clonal *P aeruginosa* and, in a small number of patients, for other non-fermentative Gram-negative organisms including *B cenocepacia*. We do not know the ideal site of deposition in the respiratory tract for *P aeruginosa* to establish infection in patients with CF, and either large or small droplets or both may be important in the pathogenesis.

Until relatively recently cross-infection with *P aeruginosa* was believed to be uncommon and limited to siblings with CF and cohorts attending the same residential CF camps.^30^ The identification of genetically related *P aeruginosa* strains in many CF centres in the UK, Europe and Australia has suggested cross-infection between patients.^23^ ^31^ Clonal strains of *P aeruginosa* contaminating the air close to patients with the same infection during physiotherapy or lung function testing have been reported.^9^ Our results provide further evidence that cross-infection may result from direct inhalation of aerosolised bacteria.

This study demonstrates widely varying bacterial counts in cough aerosols with a log normal distribution. Such a distribution is consistent with descriptions of highly infectious patients as “disseminators” (eg, in tuberculosis) or “super-spreaders” (eg, in severe acute respiratory syndrome).^32^ ^33^ Factors influencing the extent of isolation of Gram-negative bacteria in cough aerosols are likely to be complex, including both host factors and bacterial factors such as enhanced survival in air. Our data show that the concentration of bacteria in the sputum and the forced expiratory flow rates were related to cough aerosol concentration, with a trend for association with higher peak flow and higher body mass index. These data suggest that patients with milder lung disease, perhaps as a result of stronger cough, may have an increased risk of producing infectious aerosols. This warrants further investigation as the improvement in clinical outcomes in patients with CF may potentially increase the risk of spread of clonal strains of *P aeruginosa* and other Gram-negative bacteria.

The only air samples that cultured *P aeruginosa* which matched clinical samples from sputum or CASS samples were clonal AES2 strains. Given that patients who had positive air samples also had high total aerosol counts, we were unable to determine if the density of infection on its own—or whether, in addition, the nature of the specific infection—contributed to the positive air samples. The source of the *P aeruginosa* air isolates that did not match any clinical samples is unknown and environmental sampling of surfaces was not undertaken. It is possible that environmental sources such as sinks may have been involved as hand washing occurred during testing. The measured air exchange rates in the study rooms provide an important perspective as air sampling was performed for 12 min on each occasion and, during this period, 2–4 complete air exchanges occurred. Higher rates and density of positive air samples may be anticipated in less well ventilated rooms.

Nebulised hypertonic saline is now recognised as improving mucociliary clearance.^34^ We sought to determine if hypertonic saline-induced cough further enhanced the production of bacterial aerosols, but we found similar results to those seen with voluntary coughing and much greater than those obtained during tidal breathing. Treatments such as physiotherapy, mucolytic agents and even nebulised antibiotics which can induce coughing are likely to result in similar cough-induced aerosols as with voluntary coughing. Although only seen in three of seven patients tested during tidal breathing, the presence of *P aeruginosa* in the cough aerosols and in the small aerosol fraction from two patients warrants further study as any reassessment of infection control recommendations to incorporate the role of airborne transmission may not only apply to coughing patients.

The significance of aerosol-positive sputum-negative results for isolation in low numbers of *S maltophila* (n = 2) and *A xyloxidans* (n = 1) is uncertain, but it is possible that separating respiratory particles by size negates the obscuring of individual colony morphotypes by other flora which may occur with direct sputum culture.

There are several limitations to this study. First, the study was not powered to examine the effects of many of the clinical variables such as exacerbations or strain of *P aeruginosa* on the production of cough aerosols. In particular, there were few patients with unique strains of *P aeruginosa* and none who produced high concentrations of cough aerosols. The association between a specific strain and obtaining a positive air sample could not therefore be determined. Second, the media in the Andersen plates was selective for Gram-negative organisms and thus it is not possible to generalise these results to patients with CF infected with Gram-positive bacteria, mycobacteria or fungi. Third, we did not perform reproducibility or efficiency studies of the CASS. Fourth, the studies of tidal breathing were in a select group of patients who did not undertake hypertonic saline-induced cough studies and further work is required to evaluate the extent to which tidal breathing is associated with the generation of potentially infective particles. Finally, while this study provides evidence that patients with CF and Gram-negative infection can produce potentially infectious cough aerosols, we cannot draw conclusions about transmission to susceptible individuals. A recent study examined the survival of *P aeruginosa* in vitro and found bacterial survival, at least for a limited time period of <90 s, to be favoured by lower temperature and mucoid phenotype.^35^ While providing further evidence that airborne transmission is plausible, transmission by this route is yet to be proved beyond doubt.

In conclusion, this study shows that patients with CF infected with *P aeruginosa* can produce respirable infectious cough aerosols in a wide range of concentrations of a log normal distribution. We also detected other non-fermenting Gram-negative bacteria including *B cenocepacia* in the small aerosol fraction, suggesting that airborne transmission of such organisms is biologically plausible. Further studies of potential airborne transmission of bacterial pathogens in patients with CF are warranted to provide a scientific basis for infection control recommendations to prevent the spread of multidrug-resistant or clonal strains of *P aeruginosa* and other Gram-negative bacteria in this patient population.

## References

[1] Cystic Fibrosis Foundation Cystic Fibrosis Foundation Patient Registry Annual Report 2000 Bethesda, MD: Cystic Fibrosis Foundation, 2001

[2] Rosenfeld M Ramsey B Gibson R Pseudomonas acquisition in young patients with cystic fibrosis: pathophysiology, diagnosis, and management. Curr Opin Pulm Med 2003;9:492–7 14534401 10.1097/00063198-200311000-00008

[3] Pitt T Cross infection of cystic fibrosis patients with Pseudomonas aeruginosa. Thorax 2002;57:921 12403869 10.1136/thorax.57.11.921 PMC1746213

[4] Ramsey B To cohort or not to cohort: how transmissible is Pseudomonas aeruginosa? Am J Respir Crit Care Med 2002;166:906–7 12359642 10.1164/rccm.2207005

[5] Govan J Deretic V Microbial pathogenesis in cystic fibrosis: mucoid Pseudomonas aeruginosa and Burkholderia cepacia. Microbiol Rev 1996;60:539–74 8840786 10.1128/mr.60.3.539-574.1996 PMC239456

[6] Armstrong D Nixon G Carzino R Detection of a widespread clone of Pseudomonas aeruginosa in a paediatric cystic fibrosis clinic. Am J Respir Crit Care Med 2002;166:983–7 12359658 10.1164/rccm.200204-269OC

[7] Jones A Govan J Doherty C Spread of a multiresistant strain of Pseudomonas aeruginosa in an adult cystic fibrosis clinic. Lancet 2001;358:557–8 11520529 10.1016/s0140-6736(01)05714-2

[8] Doring G Jansen S Noll H Distribution and transmission of Pseudomonas aeruginosa and Burkholderia cepacia in a hospital ward. Pediatr Pulmonol 1996;21:90–100 8882212 10.1002/(SICI)1099-0496(199602)21:2<90::AID-PPUL5>3.0.CO;2-T

[9] Jones A Govan J Doherty C Identification of airborne dissemination of epidemic multiresistant strains of Pseudomonas aeruginosa at a CF centre during a cross infection outbreak. Thorax 2003;58:525–7 12775867 10.1136/thorax.58.6.525 PMC1746694

[10] Panagea S Winstanley C Walshaw MJ Environmental contamination with an epidemic strain of Pseudomonas aeruginosa in a Liverpool cystic fibrosis centre and its survival on dry surfaces. J Hosp Infect 2005;59:102–7 15620443 10.1016/j.jhin.2004.09.018

[11] Saiman L Siegal J Infection control in cystic fibrosis. Clin Microbiol Rev 2004;17:57–71 14726455 10.1128/CMR.17.1.57-71.2004 PMC321464

[12] Siegel JD Rhinehart E Jackson M 2007 guideline for isolation precautions: preventing transmission of infectious agents in health care settings. Am J Infect Control 2007;35:S65–164 18068815 10.1016/j.ajic.2007.10.007 PMC7119119

[13] Roy C Milton D Airborne transmission of communicable infection—the elusive pathway. N Engl J Med 2004;350:1710–2 15102996 10.1056/NEJMp048051

[14] Fennelly K Martyny J Fulton K Cough-generated aerosols of Mycobacterium tuberculosis: a new method to study infectiousness. Am J Respir Crit Care Med 2004;169:604–9 14656754 10.1164/rccm.200308-1101OC

[15] American Thoracic Society Standardization of spirometry, 1994 update. Am J Respir Crit Care Med 1994;152:1107–36 10.1164/ajrccm.152.3.7663792 7663792

[16] De Vos D Lim JRA Pirnay J-P Direct detection and identification of Pseudomonas aeruginosa in clinical samples such as skin biopsy specimens and expectorations by multiplex PCR based on two outer membrane lipoprotein genes, oprI and oprL. J Clin Microbiol 1997;35:1295–9 9163432 10.1128/jcm.35.6.1295-1299.1997 PMC229737

[17] Segonds C Heulin T Marty N Differentiation of Burkholderia species by PCR-restriction fragment length polymorphism analysis of the 16S rRNA gene and application to cystic fibrosis isolates. J Clin Microbiol 1999;37:2201–8 10364586 10.1128/jcm.37.7.2201-2208.1999 PMC85118

[18] Mahenthiralingam E Bischof J Byrne S DNA-based diagnostic approaches for identification of Burkholderia cepacia complex, Burkholderia vietnamiensis, Burkholderia multivorens, Burkholderia stabilis, and Burkholderia cepacia genomovars I and III. J Clin Microbiol 2000;38:3165–73 10970351 10.1128/jcm.38.9.3165-3173.2000 PMC87345

[19] Andersen A New sampler for the collection, sizing and enumeration of viable airborne particles. J Bacteriol 1958;76:471–84 13598704 10.1128/jb.76.5.471-484.1958 PMC290224

[20] Macher J Positive-hole correction of multiple-jet impactors for collecting viable microorganisms. Am Ind Hygiene J 1989;50:561–8 10.1080/15298668991375164 2688387

[21] Armstrong D Grimwood K Carlin J Lower airway inflammation in infants and young children with cystic fibrosis. Am J Respir Crit Care Med 1997;156:1197–204 9351622 10.1164/ajrccm.156.4.96-11058

[22] Lee T Brownlee K Conway S Evaluation of a new definition for chronic Pseudomonas aeruginosa infection in cystic fibrosis patients. J Cystic Fibros 2003;2:29–34 10.1016/S1569-1993(02)00141-8 15463843

[23] O’Carroll M Syrmis M Wainwright C Transmissible strains of P aeruginosa in paediatric and adult cystic fibrosis units. Eur Respir J 2004;24:101–6 15293611 10.1183/09031936.04.00122903

[24] Riley E Murphy G Riley R Airborne spread of measles in a suburban elementary school. Am J Epidemiol 1978;107:421–32 665658 10.1093/oxfordjournals.aje.a112560

[25] Frankova V Inhalatory infection of mice with influenza AO/PR8 virus. I. The site of primary virus replication and its spread in the respiratory tract. Acta Virol 1975;19:29–34 235194

[26] Riley R Mills C O’Grady F Infectiousness of air from a tuberculosis ward. Am Rev Respir Dis 1962;85:511–25 14492300 10.1164/arrd.1962.85.4.511

[27] Duguid J The size and duration of air-carriage of respiratory droplets and droplet-nuclei. J Hygiene (Lond) 1946;44:471–80 10.1017/s0022172400019288 PMC2234804 20475760

[28] Loudon R Roberts M Relation between the airborne diameters of respiratory droplets and the diameter of the stains left after recovery. Nature 1967;213:95–6

[29] Papineni R Rosenthal F The size distribution of droplets in the exhaled breath of healthy human subjects. J Aerosol Med 1997;10:105–16 10168531 10.1089/jam.1997.10.105

[30] Brimicombe RW Dijkshoorn L van der Reijden TJ Transmission of Pseudomonas aeruginosa in children with cystic fibrosis attending summer camps in The Netherlands. J Cyst Fibros 2008;7:30–6 17532271 10.1016/j.jcf.2007.04.002

[31] Scott F Pitt T Identification and characterization of transmissible Pseudomonas aeruginosa strains in cystic fibrosis patients: implications for inpatient care of respiratory patients. J Clin Microbiol 2004;53:609–15 10.1099/jmm.0.45620-0 15184530

[32] Sultan L Nyka W Mills C Tuberculosis disseminators. A study of the variability of aerial infectivity of tuberculous patients. Am Rev Respir Dis 1960;82:358–69 13835667 10.1164/arrd.1960.82.3.358

[33] Li Y Yu I Xu P Predicting super-spreading events during the 2003 severe acute respiratory syndrome epidemics in Hong Kong and Singapore. Am J Epidemiol 2004;160:719–28 15466494 10.1093/aje/kwh273 PMC7109976

[34] Elkins M Robinson M Rose B A controlled trial of long-term inhaled hypertonic saline in patients with cystic fibrosis. N Engl J Med 2006;354:229–40 16421364 10.1056/NEJMoa043900

[35] Clifton I Fletcher L Beggs C A laminar flow model of aerosol survival of epidemic and non-epidemic strains of Pseudomonas aeruginosa isolated from people with cystic fibrosis. BMC Microbiol 2008;8:105 18582388 10.1186/1471-2180-8-105 PMC2443368

